# Supplementary material for: Prothoracicostatic Activity of the Ecdysis-Regulating Neuropeptide Crustacean Cardioactive Peptide (CCAP) in the Desert Locust
Source: Int J Mol Sci. 2021 Dec 15;22(24):13465. doi: 10.3390/ijms222413465 (PMC8704491; doi:10.3390/ijms222413465)
Supplement: Supplementary file 1 [file ijms-22-13465-s001.zip › Figure S1.pdf]

Figure S1.

```
Schgr-CCAPR-1    1 -----------------------------------------------------------M
Schgr-CCAPR-2    1 -----------------------------------------------------------M
Schgr-CCAPR-3    1 -----------MGA---------------------------------------------M
Rhopr-CCAPR      1 -----------------------------------------------------------M
Drome-CCAPR      1 MLHLRLFDSSLYYTLASASESSGLASSTSTERSFNGTQGAGGVAAGGESLTPTDVAAVNL
Trica-CCAPR-1    1 -----------------------------------------------------------M
Trica-CCAPR-2    1 -----------------------------------------------------------M

Schgr-CCAPR-1    2 DDLNG-----------------------------------------TNASDAAPVINSFY
Schgr-CCAPR-2    2 DASAGGNATAS----------SLLPGDGADN----------LTADAAAANDTQEVINSFY
Schgr-CCAPR-3    5 EEEPPPAAVTSPDSEAALTSPAPSPAVVVTTTAVPDWSTTDFSGLLYTAATSGEDINSFY
Rhopr-CCAPR      2 DWVIRDNYSNP----------------------------------AANITNTTDEINSFY
Drome-CCAPR     61 TYFTPAISHVMLAPTTIATTTASATMVQIQTTAAPSHDLETGGNSTSSDPGEFDNLNSFY
Trica-CCAPR-1    2 SYIEDAN----------------------------------------ETDSNNTSLDAFY
Trica-CCAPR-2    2 SGAPIPDVGQS-----WLKSNSSLAGFEVNS-----------SEQLTTPTPT-DINSFY

                                TM-1                             TM-2
Schgr-CCAPR-1   21 FYETEQFAVLWSLFTLIVVGNAAVLVTLLAGKRRKSRMNFFIVQLALADLSVGLISVLTD
Schgr-CCAPR-2   42 FYETEQFTVLWILFASIVLGNAAVLVALLVNKARKSRMNFFIMHLALADLSVGVISVLTD
Schgr-CCAPR-3   65 FYQTEQLTVLWILFALIVLGNSAVLIALYLNKNRKSRMNFFIMQLALADLTVGLINVLTD
Rhopr-CCAPR     28 FYQTEQFTVLWLLFAAIVLGNSAVLLALLFNKSSKSRMNFFIMHLAFADLSVGLISVLTD
Drome-CCAPR    121 FYETEQFAVLWILFTVIVLGNSAVLFVMFINKNRKSRMNYFIKQLALADLCVGLINVLTD
Trica-CCAPR-1   22 FFETEQFTLLWVLFVIIVAGNVGVLYTLLFGRSRKSRMNYFITHLALADLSVGLINVLTD
Trica-CCAPR-2   44 FYKTEQFTVLWVLFTMIVLGNSAVLVALLVSKSRKSRMNFFICQLAIADLAVGLISVSTD

                             *         TM-3
Schgr-CCAPR-1   81 IVWRMTVAWNAGNAACKLIRFLQAVVTYSSTYVLVALSIDRYDAITHPMNFSGSWRRARL
Schgr-CCAPR-2  102 IVWRMTVSWNAGNAACKIIRFLQVVVTYSSTYVLVALSIDRYDAITHPMNFSGSWRRARL
Schgr-CCAPR-3  125 IVTRITIVWYAGNVACKIVRFLQAVVTYSSTYMLVALSIDRYDAITHPMNFSGSWRRARA
Rhopr-CCAPR     88 IIWRITVEWKAGNVVCKVVRFMQAVVTYSSTYVLVALSIDRLDAITRPMNFSGSWRRARL
Drome-CCAPR    181 IIWRITISWRAGNLACKAIRFSQVCVTYSSTYVLVAMSIDRYDAITHPMNFSKSWKRARH
Trica-CCAPR-1   82 IIWKTTVAWYAGNVACKVIRFLQVVVTYASTYVLVALSIDRYDAIRHPMKFSGSWKRARY
Trica-CCAPR-2  104 IAWRITVAWYAGNVLCKIIRFLQAVVTYSSTYVLVALSIDRYDAITHPMNFSGSWKRARV

                         TM-4                  *                 TM-5
Schgr-CCAPR-1  141 LVAAAWLLSAVFSVPIAVLYHERPVEGRLQCWIDFPQAWQWQLYMTLVALTLFALPAVII
Schgr-CCAPR-2  162 LVLSAWLLSFLFSVPIIIFYEEKTIQGHPQCWIDFQERWQWRLYMTLVTTTLFVLPALII
Schgr-CCAPR-3  185 LVVTAWGLSFLFSIPSMIFFEEQPIQGHAQCWIDFPHNWQWQVYMTLVAVALFIVPTVII
Rhopr-CCAPR    148 LVGFSWALSAFFSSPILILYKERLIQGSFQCWIELGSTLKWQIYMSLVAVSLFLVPALVI
Drome-CCAPR    241 LVAGAWLISALFSLPILVLYEEKLIQGHPQCWIELGSPIAWQVYMSLVSATLFAIPALII
Trica-CCAPR-1  142 LIIAAWFFSALYSLPILIFYEEKIVKGKLQCWIEFHPQWKWQVYMTLVSLSLFIIPASII
Trica-CCAPR-2  164 LVMLAWLLSILFSLPTVFLFEEKQVQSMPQCWIDLQT-WQWKVYITLVALVLFVFPALII

Schgr-CCAPR-1  201 SACYTVIVSTIWSKGRHMAPPPPYTDARTGSCRPHQLLHQHSHSRLSSGKSRDDEADGRR
Schgr-CCAPR-2  222 TACYTVIVLTIWSKSKVLTPPSRLK---------------NGDHRSG------DDHDSRR
Schgr-CCAPR-3  245 SACYAVIVFTIWSKSKLLAPACGRPG---------GNSRAGSGVSRGPAPSFSDDQDCRR
Rhopr-CCAPR    208 TACYTVIVYTIWTKSIHISRDSQQST------------------PLKNGGDKGDDNDIRR
Drome-CCAPR    301 SACYAIIVKTIWAKGSIFVPTER---------------------------AGFGAAPARR
Trica-CCAPR-1  202 ATCYAIIIITIWSKN-------A-------------------KGFIKNTKAVNGSDNSRR
Trica-CCAPR-2  223 SACYAVIVRTIWSKSKLLIPVGHIPI------------RQCDDHRDRPPRHFHEEHDTRR

                                      TM-6
Schgr-CCAPR-1  261 ASSRGIIPKAKVKTVKMTFVIVFMFVVCWSPYIVFDLLQVYGQVPRTQANIAVASFIQSL
Schgr-CCAPR-2  261 ASSRGLIPKAKIKTVKMTFVIVFVFILCWSPYIVFDLLQVYDHVPTTQTNIAVASFIQSL
Schgr-CCAPR-3  296 ASSRGLIPRAKIKTVKMTFVIVFVFVLCWSPYFVFDLLQVFGHVPRTQTMVALATFIQSL
Rhopr-CCAPR    250 ASSRGIIPRAKIKTVKMTFVIVFVFILCWSPYIVFDLLQVFGYVPRTQTNIAVATFIQSL
Drome-CCAPR    334 ASSRGIIPRAKVKTVKMTLTIVFVFIICWSPYIIFDLLQVFGQIPHSQTNIAIATFIQSL
Trica-CCAPR-1  236 ASSRGLIPRAKVKTIKITFVIVSVFILCWSPYIIFDLLQVFGQIPGTQTNIAIATFIQSL
Trica-CCAPR-2  271 ASSRGIIPRAKIKTVKMTLVIVFVFVLCWSPYIVFDLLQVYGHIPKTQTNIAIATLIQSL
```

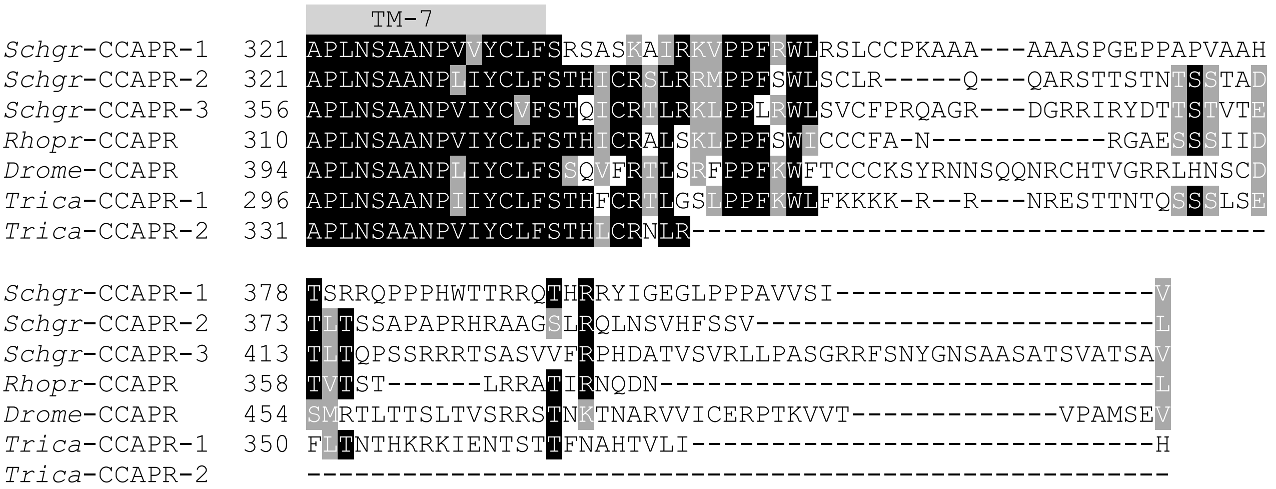

Multiple sequence alignment of *Schgr*-CCAPRs with other insect CCAPRs. Identical residues between the aligned sequences are highlighted in black, and conservatively substituted residues in grey. Amino acid position is indicated at the left and dashes indicate gaps that are introduced to maximize similarities in the alignment. Putative transmembrane regions (TM1-5) of *Schgr*-CCAPR-1, -2 and -3 are indicated by dark grey bars and putative transmembrane regions (TM6-7) of *Schgr*-CCAPR-1 and -2 are indicated by light grey bars. Conserved residues that are predicted to form disulphide bridges in extracellular loop 1 and 2 are indicated (*). Rhodopsin-like DRY motif is boxed in a red dotted line. NXS/T consensus sequences for putative N-glycosylation sites are underlined in red. Abbreviations: CCAPR = crustacean cardioactive peptide receptor, *Schgr* = *Schistocerca gregaria*, *Rhopr* = *Rhodnius prolixus* (AGT02811.1), *Drome* = *Drosophila melanogaster* (AAO66429), *Trica* = *Tribolium castaneum* (1:ABN79651, 2:ABN79652)
