# Supplementary material for: Prothoracicostatic Activity of the Ecdysis-Regulating Neuropeptide Crustacean Cardioactive Peptide (CCAP) in the Desert Locust
Source: Int J Mol Sci. 2021 Dec 15;22(24):13465. doi: 10.3390/ijms222413465 (PMC8704491; doi:10.3390/ijms222413465)
Supplement: Supplementary file 1 [file ijms-22-13465-s001.zip › Figure S2.pdf]

Figure S2.

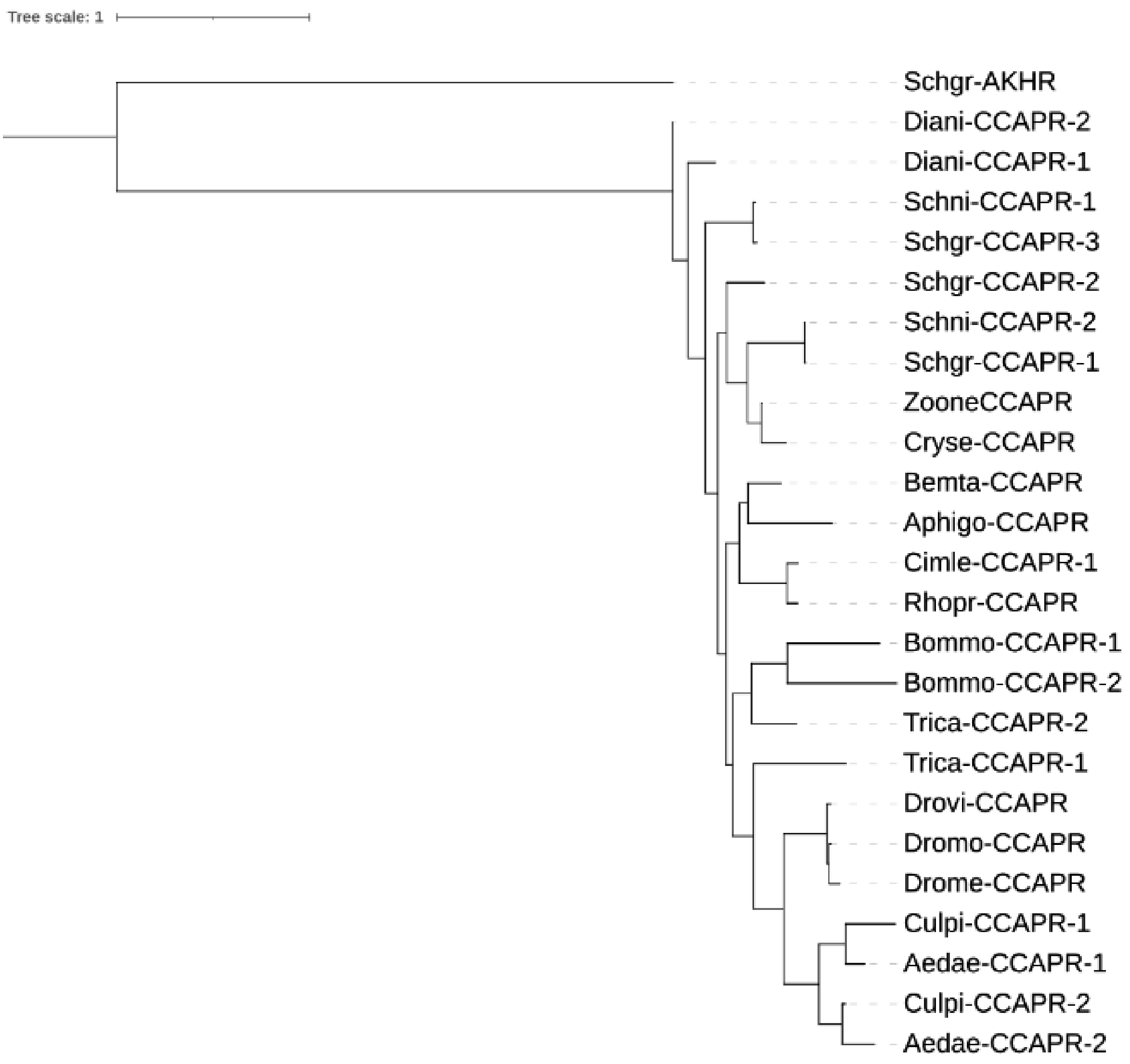

Phylogenetic analysis of CCAPR orthologs from insect species. CCAPR sequences were aligned with MUSCLE and used for a maximum likelihood analysis using IQ-tree. The resulting tree was rooted using the *Schgr* -AKHR sequence as the outgroup. Scale values indicate evolutionary distance. Tree was visualized with iTOL. Abbreviations: *Schgr* = *Schistocerca gregaria* (1:MZ647657, 2:MZ647658, 3:MZ647659), *Diani* = *Dianemobius nigrofaciatus* (1:IADE01117080.1, 2: :IADE01117083.1), *Schni* = *Schistocerca nitens* (1: GIOW01102367.1, 2: GIOW01112628.1), *Cryse* = *Cryptotermes secundus* (XM_033755399.1), *Zoone* = *Zootermopsis nevadensis* (XP_021921974.1), *Aphgo* = *Aphis gossypii* (XM_027997733.1), *Bemta* = *Bemisia tabaci*, *Rhopr* = *Rodnius prolixus* (AGT02811.1), *Cimle* = *Cimex lectularius* (1:XP_014248905.2, 2:XP_014248903.1), *Bommo* = *Bombyx mori* (1:NP_001127724.1, 2:NP_001127746.1), *Trica* = *Tribolium castaneum* (1:ABN79651, 2:ABN79652), *Drovi* = *Drosophila virilis* (GJ23325), *Drome* = *Drosophila melanogaster* (AAO66429), *Dromo* = *Drosophila mojavensis* (GI22912), *Aedae* = *Aedes aegypti* (1:AGAP001961, 2: XP_321100.4) and *Culpi* = *Culex pipiens* (1:CPIJ006268, 2:XP_001847670.1).
