# Supplementary material for: Prothoracicostatic Activity of the Ecdysis-Regulating Neuropeptide Crustacean Cardioactive Peptide (CCAP) in the Desert Locust
Source: Int J Mol Sci. 2021 Dec 15;22(24):13465. doi: 10.3390/ijms222413465 (PMC8704491; doi:10.3390/ijms222413465)
Supplement: Supplementary file 1 [file ijms-22-13465-s001.zip › Figure S3.pdf]

Figure S3.

```
                                 Signal peptide                             CCAP
Schgr-CCAPpre    1 --MSRALLMLCPALLVLLACLQRAAADDVIMEKRDMDSPFLNRLFEAKMKRPFCNAFTGC
Rhopr-CCAPpre    1 ------MQLLVPCFLLFTALVSAVLTDDVFLQKRLYFPGEIAEPIDPKMKKPFCNAFTGC
Drome-CCAPpre    1 MRTSMRISLRLLALLACAICSQASLERENNEGTNMANHKLSGVIQWKYEKRPFCNAFTGC
Manse-CCAPpre    1 MTVSRVCLLLLVALVYLDCCYAASIP-------RNFDPRLSEEIVMAPKKRPFCNAFTGC
Bommo-CCAPpre    1 M-TSRVLLVLVVALLCAECCVTATIP-------RNFDPRSNEEMVTMPKKRPFCNAFTGC

Schgr-CCAPpre   59 GKKRSD---------------------------ESVGTLLEMNSEPAVADLSRQILSEAK
Rhopr-CCAPpre   55 GKKRSD---------------------------ESMATLVDLNSEPAVEELSRQILSEAK
Drome-CCAPpre   61 GRKRTY--PSYPPFSLFKRNEVEEKPYNNEYLSEGLSDLIDINAEPAVENVQKQIMSQAK
Manse-CCAPpre   54 GRKRSQGPPGMPAQDL-RTKQYLDE--------EALGSI--LDSESAIDELSRQILSEAK
Bommo-CCAPpre   53 GRKRSQTAPGMPNQDLMRQRQYVDE--------DTLGTM--LDSESAIDELSRQILSEAK
                   -

Schgr-CCAPpre   92 LWEAIQEARAELLARRRQHEMQTNRLADFSRPLAVAQYRKKRAAAPPTPVQGMKPWRR
Rhopr-CCAPpre   88 LWEAIQEARMELLNRKQQQ---SDRIP--LQPLPLTTIRKRS-HYLY----------T
Drome-CCAPpre  119 IFEAIKEASKEIFRQKNKQKMLQNE-----KEM--QQLEERE-S-------------K
Manse-CCAPpre  103 LWEAIQEASAEIARRKQKEAYI-----------------------------------Q
Bommo-CCAPpre  103 LWEAIQEASAEIARRKQKEFYNS----------------------------------Q
```

Multiple sequence alignment of insect CCAP precursors. Identical residues between the aligned sequences are highlighted in black, and conservatively substituted residues in grey. Amino acid position is indicated at the left and dashes indicate gaps that are introduced to maximize similarities in the alignment. The signal peptide and CCAP peptide sequence are indicated by dark grey bars. Abbreviations: CCAPpre = crustacean cardioactive prepropeptide, *Schgr* = *Schistocerca gregaria*, *Rhopr* = *Rhodnius prolixus* (ADK73625.1), *Drome* = *Drosophila melanogaster* (AGB96226.1), *Manse* = *Manduca sexta* (AAL39064.1), *Bommo* = *Bombyx mori* (BAG50376.1).
