# Supplementary material for: Prothoracicostatic Activity of the Ecdysis-Regulating Neuropeptide Crustacean Cardioactive Peptide (CCAP) in the Desert Locust
Source: Int J Mol Sci. 2021 Dec 15;22(24):13465. doi: 10.3390/ijms222413465 (PMC8704491; doi:10.3390/ijms222413465)
Supplement: Supplementary file 1 [file ijms-22-13465-s001.zip › Figure S4.pdf]

**Figure S4**

**A**

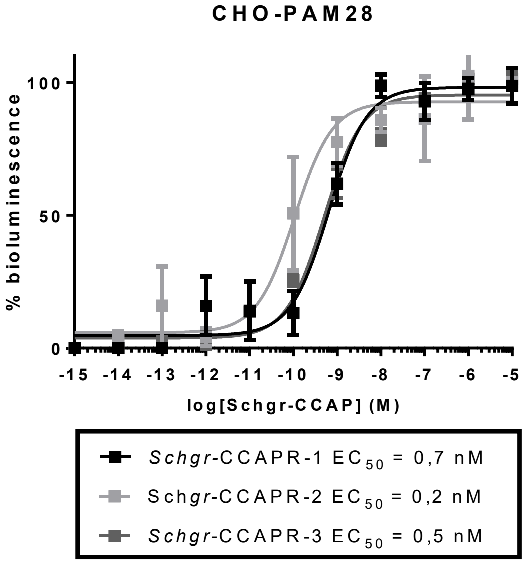


**B**

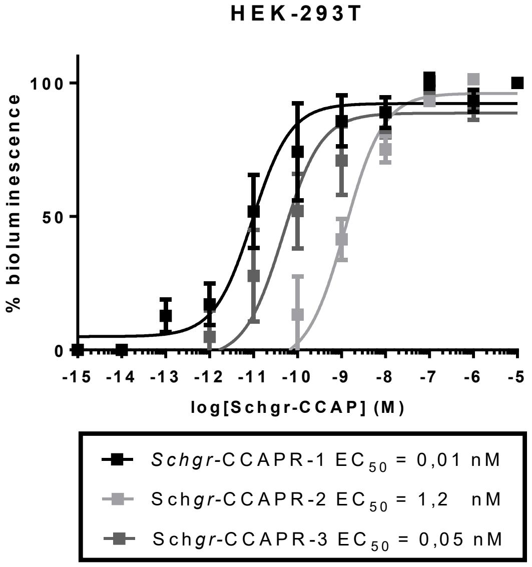


**Dose-response curves induced by CCAP for cell-based receptor activity assays A) in CHO-PAM28-*Schgr*-CCAPR-1, -2 or -3 cells and B) in HEK293T *Schgr*-CCAPR-1, -2 or -3 cells, co-transfected with $CRE_{(6x)}$-Luc construct.** These cell-based receptor activity data were obtained from three independent experiments. The bioluminescence was measured in triplicate per concentration of *Schgr*-CCAP and per transfection. Error bars represent the S.E.M and the 100 % level refers to the maximal response level. The zero-response level corresponds to treatment with BSA medium only. In addition, the half maximal effective concentrations ($EC_{50}$ values) are indicated. Abbreviations used: *Schgr* = *Schistocerca gregaria*, CCAPR = crustacean cardioactive peptide receptor, CHO-PAM28 = chinese hamster ovary PAM28 cells and HEK-293T = human embryonic kidney 293 T cells.
