# Supplementary material for: Prothoracicostatic Activity of the Ecdysis-Regulating Neuropeptide Crustacean Cardioactive Peptide (CCAP) in the Desert Locust
Source: Int J Mol Sci. 2021 Dec 15;22(24):13465. doi: 10.3390/ijms222413465 (PMC8704491; doi:10.3390/ijms222413465)
Supplement: Supplementary file 1 [file ijms-22-13465-s001.zip › Figure S5.pdf]

Figure S5

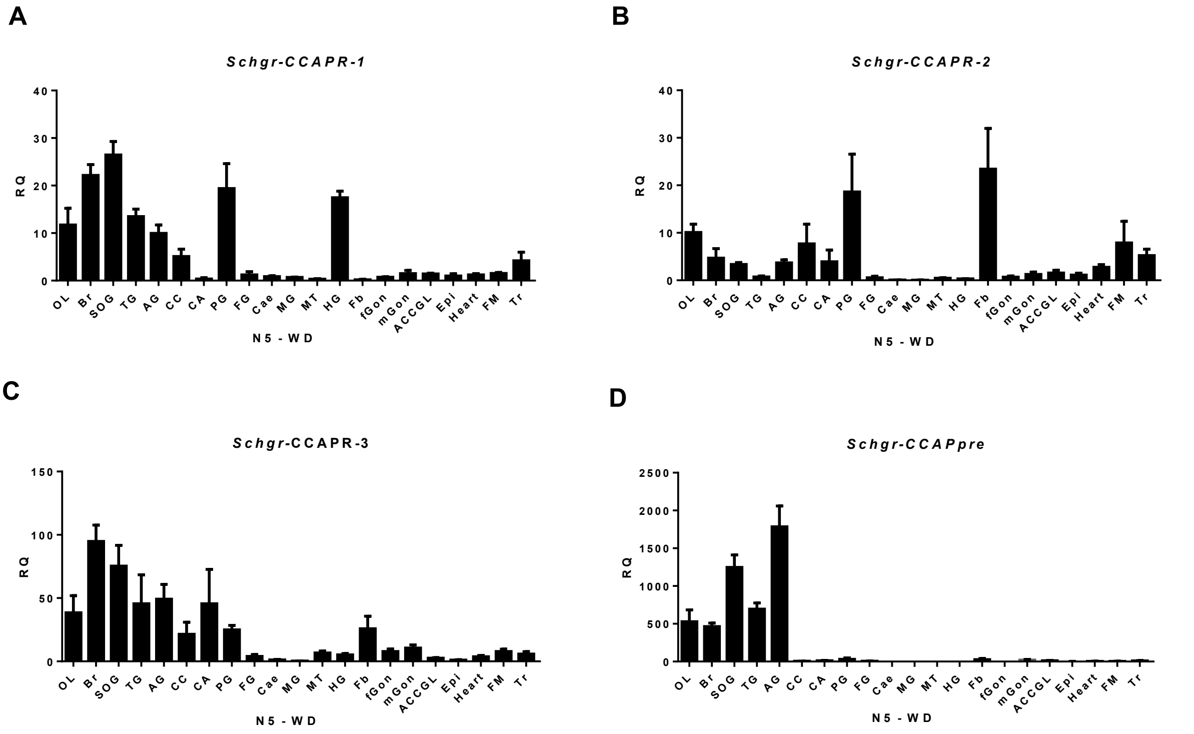

Tissue distribution of CCAP precursor and receptor transcripts in fifth nymphal (N5) *S. gregaria* at the molt cycle moment of weight decrease (WD). A) Transcript profile of *Schgr-CCAPR-1*, B) *Schgr-CCAPR-2*, C) *Schgr- CCAPR-3* and D) *Schgr-CCAPpre*. The data represent mean values ±S.E.M. of three independent pools of five animals, run in duplicate and normalized relative to *EF1α* and *RP49* reference gene transcript levels. Abbreviations used: OL = optic lobes, Br = brain, SOG = suboesophageal ganglion, TG = thoracic ganglia, AG = abdominal ganglia, CC = corpora cardiaca, CA = corpora allata; PG = prothoracic glands, FG = foregut, Cae = caeca, MG = midgut, MT = Malpighian tubules HG = hindgut, Fb = fat body, fGon = female gonads, mGon = male gonads, ACCGL = male accessory glands, Epi = epidermis, FM = flight muscle and Tr = Trachea.
