# Supplementary material for: Prothoracicostatic Activity of the Ecdysis-Regulating Neuropeptide Crustacean Cardioactive Peptide (CCAP) in the Desert Locust
Source: Int J Mol Sci. 2021 Dec 15;22(24):13465. doi: 10.3390/ijms222413465 (PMC8704491; doi:10.3390/ijms222413465)
Supplement: Supplementary file 1 [file ijms-22-13465-s001.zip › Figure S6.pdf]

Figure S6

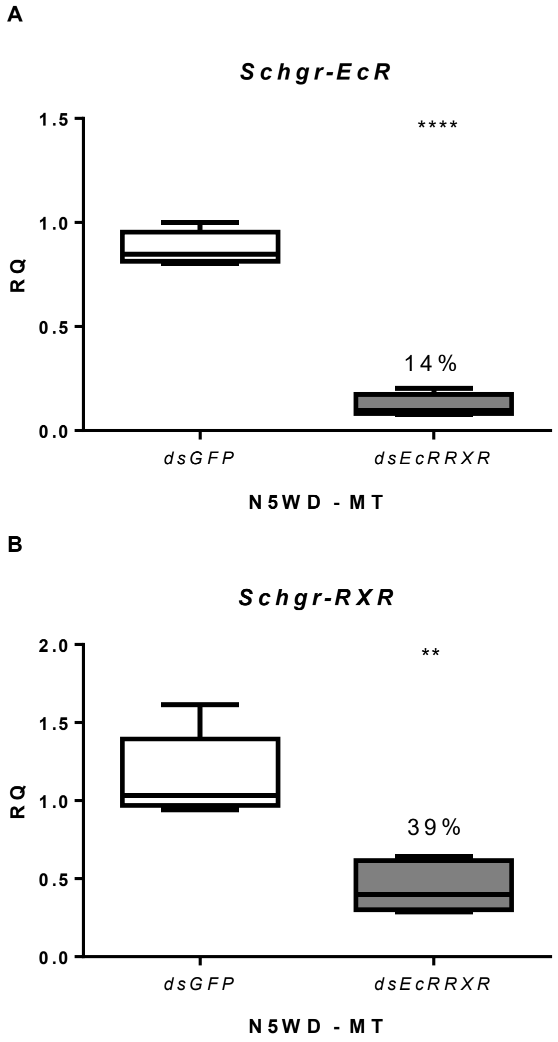

Effect of the RNAi-mediated knockdown on A) *Schgr-EcR* and B) *Schgr-RXR* transcript levels in Malpighian tubules as caused by *dsGFP*- and *dsEcR/RXR*-injections in fifth instar nymphs at weight decrease. *dsRNA* constructs were injected at N5D0. A boost injection was administered at N5D3. Significant differences ($p<0.01$ and $p<0.0001$) are indicated by asterisks (** and ****, respectively) and were calculated by an unpaired Student's T-Test.
