# Supplementary material for: Prothoracicostatic Activity of the Ecdysis-Regulating Neuropeptide Crustacean Cardioactive Peptide (CCAP) in the Desert Locust
Source: Int J Mol Sci. 2021 Dec 15;22(24):13465. doi: 10.3390/ijms222413465 (PMC8704491; doi:10.3390/ijms222413465)
Supplement: Supplementary file 1 [file ijms-22-13465-s001.zip › Figure S7.pdf]

Figure S7

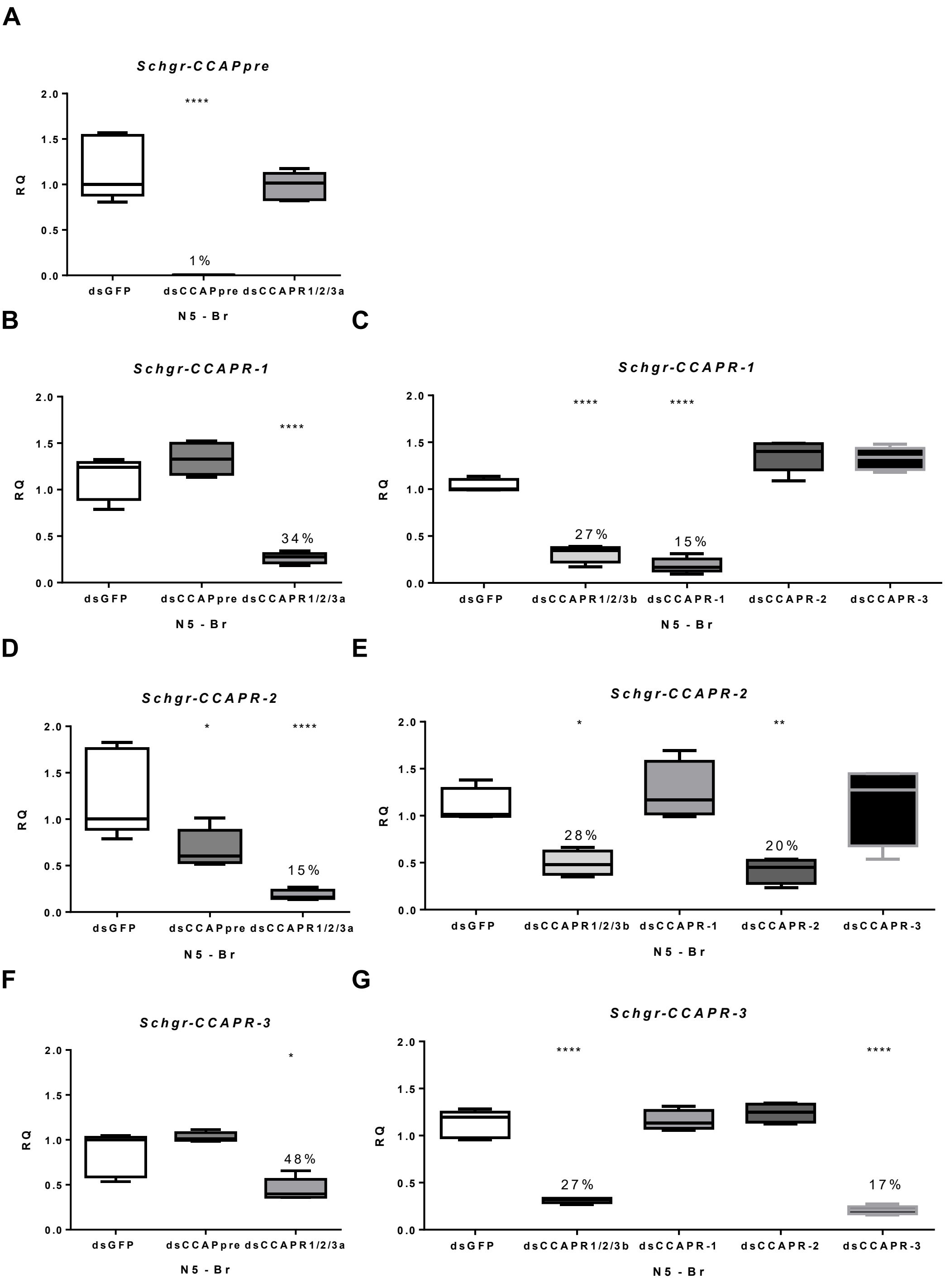

Effect of the RNAi-mediated knockdown on *Schgr-CCAPpre* and *Schgr-CCAPR-1*, *-2* and-*3* transcript levels in brain as caused by *dsGFP-, dsCCAPpre-, dsCCAPR1/2/3a-, dsCCAPR1/2/3b, dsCCAPR-1, dsCCAPR-2* or *dsCCAPR-3*-injections in fifth instar (N5) *S. gregaria* nymphs. A) Relative quantities of *Schgr-CCAPpre* transcript levels in *dsGFP*, *dsCCAPpre* and *dsCCAPR1/2/3a* treated nymphs. B) Relative quantities of *Schgr-CCAPR-1* transcript levels in *dsGFP*, *dsCCAPpre* and *dsCCAPR1/2/3a* treated nymphs and in C) *dsGFP*, *dsCCAPR1/2/3b*, *dsCCAPR-1*, *dsCCAPR-2* and *dsCCAPR-3* treated nymphs. D) Relative quantities of *Schgr-CCAPR-2* transcript levels in *dsGFP*, *dsCCAPpre* and *dsCCAPR1/2/3a* treated nymphs and in E) *dsGFP*, *dsCCAPR1/2/3b*, *dsCCAPR-1*,

dsCCAPR-2 and *dsCCAPR-3* treated nymphs. F) Relative quantities of *Schgr-CCAPR-3* transcript levels in *dsGFP*, *dsCCAPpre* and *dsCCAPR1/2/3a* treated nymphs and in G) *dsGFP*, *dsCCAPR1/2/3b*, *dsCCAPR-1*, *dsCCAPR-2* and *dsCCAPR-3* treated nymphs. *dsRNA* constructs were injected at N4D0. Boost injections were administered at N4D3, N5D1, N5D4. Significant differences ($p<0.05$ and $p<0.0001$) are indicated by asterisks (* and ****, respectively) and were calculated by One-way ANOVA, followed by a Dunnett's Test.
