# Supplementary material for: Prothoracicostatic Activity of the Ecdysis-Regulating Neuropeptide Crustacean Cardioactive Peptide (CCAP) in the Desert Locust
Source: Int J Mol Sci. 2021 Dec 15;22(24):13465. doi: 10.3390/ijms222413465 (PMC8704491; doi:10.3390/ijms222413465)
Supplement: Supplementary file 1 [file ijms-22-13465-s001.zip › Figure S8.pdf]

Figure S8

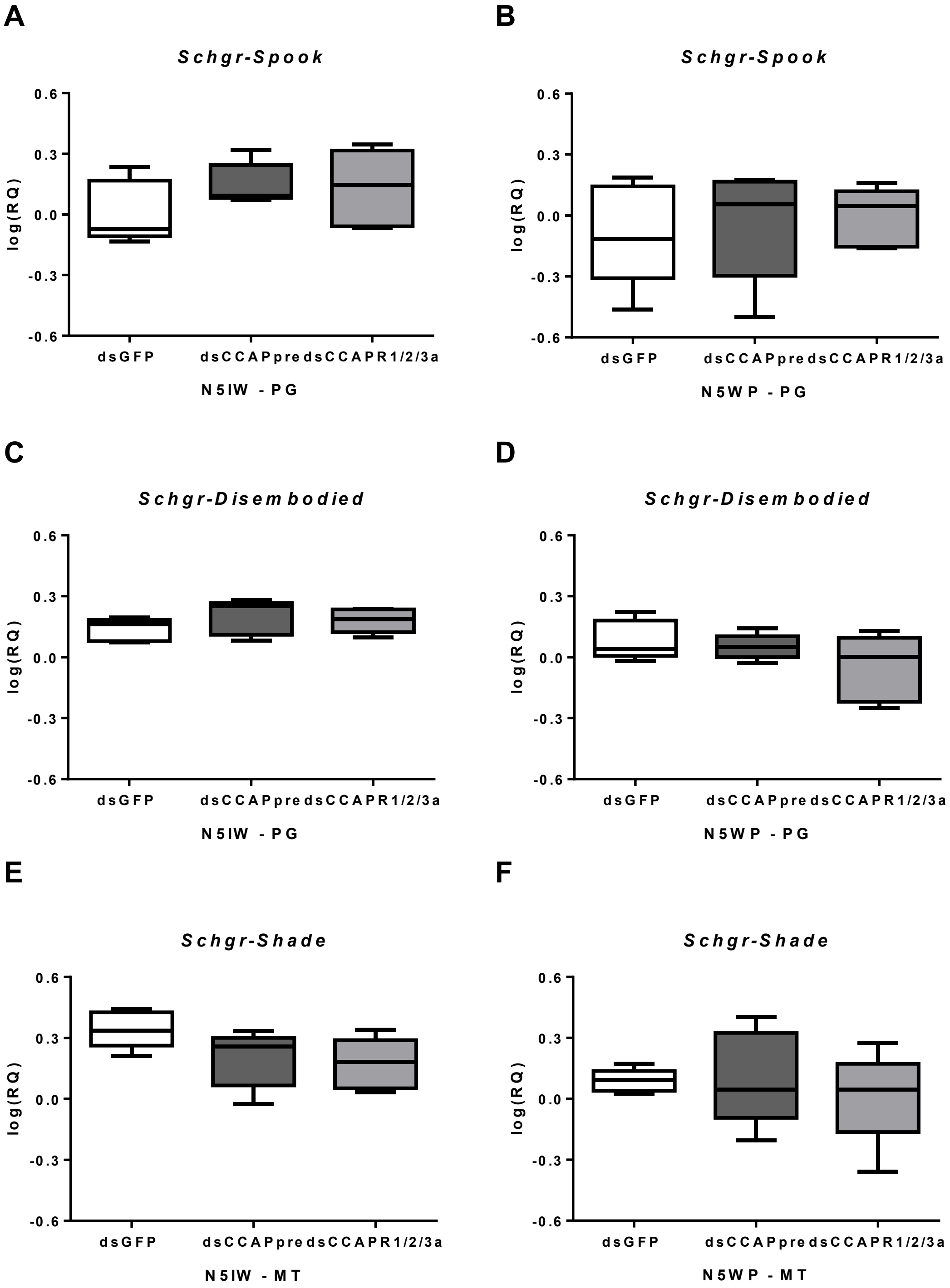

Effects of *dsGFP-, dsCCAPpre-* or *dsCCAPR1/2/3a*-injections on the expression of the Halloween genes in nymphs of the fifth nymphal stage (N5) at the molt cycle moments, IW (inter-wing distance of at least 1.8 mm) and WP (weight peak). *A) Schgr-Spook transcript* levels in prothoracic glands (PG) at N5IW and B) at N5WP. C) *Schgr-Disembodied* transcript levels in PG at N5IW and D) at N5WP and I) at N5WD. E) *Schgr-Shade* transcript levels in Malpighian tubules (MT) at N5IW and F) at N5WP. The box plots (min to max) represent data obtained from 5 independent pools of three locusts, run in duplicate. One-way ANOVA, followed by a Dunnett's Test, on log-transformed data, was performed as statistical analysis.
