# Supplementary material for: Prothoracicostatic Activity of the Ecdysis-Regulating Neuropeptide Crustacean Cardioactive Peptide (CCAP) in the Desert Locust
Source: Int J Mol Sci. 2021 Dec 15;22(24):13465. doi: 10.3390/ijms222413465 (PMC8704491; doi:10.3390/ijms222413465)
Supplement: Supplementary file 1 [file ijms-22-13465-s001.zip › Table S1.pdf]

Table S1. Structure of the peptides used in current study.

Peptide	Amino acid sequence
Schgr-CCAP	PF**C**NAFTG**C**amide
Schgr-CHHa2.1	G**C**MAFGHS**C**FGGHamide
Schgr-CHHa2.2	AGG**C**MAFGHS**C**FGGHamide
Schgr-ASTC	pQRKPRYYR**C**YFNPIS**C**Famide
Schgr-ASTCCC	SYWKQ**C**AFNAVS**C**Famide
Schgr-Crz	pQTFQYSHGWTNamide
Schgr-AKH-I	pQLNFTPNWGTamide
Schgr-AKH-II	pQLNFSTGWamide
Schgr-INT	**C**LITN**C**PRGamide

The cysteine residues that form a disulphide bridge are depicted in a red bold "C" letter. Abbreviations used: *Schgr* = *Schistocerca gregaria*; CCAP = crustacean cardioactive peptide; CHHa = CHHamide; ASTC = allatostatin C; ASTCCC = Allatostatin CCC; Crz = corazonin; AKH-I = adipokinetic hormone I; AKH-II = adipokinetic hormone II; INT = inotocin.
