# Supplementary material for: Prothoracicostatic Activity of the Ecdysis-Regulating Neuropeptide Crustacean Cardioactive Peptide (CCAP) in the Desert Locust
Source: Int J Mol Sci. 2021 Dec 15;22(24):13465. doi: 10.3390/ijms222413465 (PMC8704491; doi:10.3390/ijms222413465)
Supplement: Supplementary file 1 [file ijms-22-13465-s001.zip › Table S2.pdf]

Table S2.

Oligonucleotide sequences of primers used in cloning and sequence analysis of *Schgr-CCAPpre*, *Schgr-CCAPR-1*, *Schgr-CCAPR-2* and *Schgr-CCAPR-3*.

	**Forward primer**	**Reverse primer**
Schgr-CCAPpre	ACCCCCACACAGGAAGTACA	ACAGTACAGGGCAGGGTGAG
Schgr-CCAPpre-nested	ATGTCACGAGCCCTGCTGATG	CTACCGGCGCCACGGCTT
Schgr-CCAPR-1	ACCCATGGACGACCTCAA	TGTGGAGAAGAGGATGATTCC
Schgr-CCAPR-1-nested	CACCATGGACGACCTCAATGGCA	CTAGACGATGGAGACGACAGC
Schgr-CCAPR-2	GTGCGTGAGTGCGTGA	CGTACTCGTGCAGCTAGAGCAC
Schgr-CCAPR-2-nested	CACCATGGACGCCAGCGC	CTAGAGCACGGACGAGAA
Schgr-CCAPR-3	CCCGTCCATTGTTACCTGAC	TGACGTCGTAACATCGTCGT
Schgr-CCAPR-3-nested	CCACATGGGGCCATGGAG	CTGCGTCAGACGGCCGACGTG

Abbreviations: *Schgr*: *Schistocerca gregaria CCAPpre* = crustacean cardioactive peptide precursor, *CCAPR* = crustacean cardioactive peptide receptor. A partial Kozak sequence (CACC) was added to the 5' end of the forward primers of *Schgr-CCAPR-1-nested*, *Schgr-CCAPR-2-nested*, *SchgrCCAPR-3-nested* to allow initiation of translation in mammalian cells.
