# Supplementary material for: Prothoracicostatic Activity of the Ecdysis-Regulating Neuropeptide Crustacean Cardioactive Peptide (CCAP) in the Desert Locust
Source: Int J Mol Sci. 2021 Dec 15;22(24):13465. doi: 10.3390/ijms222413465 (PMC8704491; doi:10.3390/ijms222413465)
Supplement: Supplementary file 1 [file ijms-22-13465-s001.zip › Table S3.pdf]

Table S3

Oligonucleotide sequences for primers used in *dsRNA* construct design. Underlined sequences are the T7 promoter sequences.

dsRNA construct	Forward primer	Reverse primer
dsCCAPpre	5'- TAATACGACTCACTATAGGGAGA GGCACTCTGCTGGAGATGA-3'	5'-TAATACGACTCACTATAGGGAGA CAGTGGGCGAGAGAAGTCTG-3'
dsCCAPR1/2/3a	5'-TAATACGACTCACTATAGGGAGA CTACGAGACGGAGCAGTTC-3'	5'-TAATACGACTCACTATAGGGAGA ATGGCGTCGTAGCGATCTAT-3'
dsCCAPR1/2/3b	5'-TAATACGACTCACTATAGGGAGA AGCTGTACATGACGCTGGTG-3'	5'- TAATACGACTCACTATAGGGAGA CCACGAACATGAAGACGATG-3'
dsCCAPR-1	5'-TAATACGACTCACTATAGGGAGA TCGCTGTGCTGCCCCAA-3'	5'-TAATACGACTCACTATAGGGAGA CTAGACGATGGAGACGACAGCT-3'
dsCCAPR-2	5'-TAATACGACTCACTATAGGGAGA CTACTGCCTCTTCTCCACGC-3'	5'- TAATACGACTCACTATAGGGAGA CCTCAGAAGTGCACCCTGTT-3'
dsCCAPR-3	5'-TAATACGACTCACTATAGGGAGA TCACCAGCCCCGACTCGGAG-3'	5'-TAATACGACTCACTATAGGGAGA AGGTGGCTGCAGTGTAGAGGA-3'
dsGFP	5'-TAATACGACTCACTATAGGGAGA AAGGTGATGCTACATACGGAA-3'	5'-TAATACGACTCACTATAGGGAGA ATCCCAGCAGCAGTTACAAAC-3'

Abbreviations: *CCAPpre = crustacean cardioactive peptide precursor, CCAPR = crustacean cardioactive peptide receptor, GFP = green fluorescent protein*
