# Supplementary material for: Prothoracicostatic Activity of the Ecdysis-Regulating Neuropeptide Crustacean Cardioactive Peptide (CCAP) in the Desert Locust
Source: Int J Mol Sci. 2021 Dec 15;22(24):13465. doi: 10.3390/ijms222413465 (PMC8704491; doi:10.3390/ijms222413465)
Supplement: Supplementary file 1 [file ijms-22-13465-s001.zip › Table S4.pdf]

Table S4

Oligonucleotide sequences for primers used in qRT-PCR.

Reference genes	Forward primer	Reverse primer
CG13220	5’-TGTTCAGTTTTGGCTCTGTTCTGA-3’	5’- ACTGTTCTCCGGCAGAATTGC-3’
EF1α	5’-GATGCTCCAGGCCACAGAGA-3’	5’-TGCACAGTCGGCCTGTGAT-3’
RP49	5’-CGCTACAAGAAGCTTAAGAGGTCAT-3’	5’-CCTACGGCGCACTCTGTTG-3’
GAPDH	5’-GTCTGATGACAACAGTGCAT-3’	5’-GTCCATCACGCCACAACTTTC-3’
α-Tub	5’- TGACAATGAGGCCATCTATG-3’	5’- CGCAAAGATGCTGTGATTGA-3’

Target genes	Forward primer	Reverse primer
Schgr-CCAPpre	5’-AAAATGAAGAGACCCTTCTGCAA-3’	5’-TCTCGTCCGACCGCTTCT-3’
Schgr-CCAPR-1	5’-GCTGTCGTCTCCATCGTCTA-3’	5’-GAAGAAGCCGCTCGAATTGG-3’
Schgr-CCAPR-2	5’-CCTTTCGTGACGCCTCTGA-3’	5’- CCGAAATTCATTGCCCACTGT-3’
Schgr-CCAPR-3	5’-TCTGGCCATCTGTCTTTCT-3’	5’- ATTCTTGTTGGCCGCTGTTG-3’

Abbreviations: EF1*α = elongation factor 1 alpha, RP49 = ribosomal protein 49, GAPDH = Glyceraldehyde-3-phosphate dehydrogenase, α-Tub= α-Tubulin, CCAPpre = crustacean cardioactive peptide precursor, CCAPR = crustacean cardioactive peptide receptor.*
